# Diagnostic approach to peripheral neuropathy

**DOI:** 10.4103/0972-2327.41875

**Published:** 2008

**Authors:** Usha Kant Misra, Jayantee Kalita, Pradeep P. Nair

**Affiliations:** Department of Neurology, Sanjay Gandhi Post Graduate Institute of Medical Sciences, Lucknow, India

**Keywords:** Axonal demyelination, diagnosis, nerve conduction, peripheral neuropathy

## Abstract

Peripheral neuropathy refers to disorders of the peripheral nervous system. They have numerous causes and diverse presentations; hence, a systematic and logical approach is needed for cost-effective diagnosis, especially of treatable neuropathies. A detailed history of symptoms, family and occupational history should be obtained. General and systemic examinations provide valuable clues. Neurological examinations investigating sensory, motor and autonomic signs help to define the topography and nature of neuropathy. Large fiber neuropathy manifests with the loss of joint position and vibration sense and sensory ataxia, whereas small fiber neuropathy manifests with the impairment of pain, temperature and autonomic functions. Electrodiagnostic (EDx) tests include sensory, motor nerve conduction, F response, H reflex and needle electromyography (EMG). EDx helps in documenting the extent of sensory motor deficits, categorizing demyelinating (prolonged terminal latency, slowing of nerve conduction velocity, dispersion and conduction block) and axonal (marginal slowing of nerve conduction and small compound muscle or sensory action potential and dennervation on EMG). Uniform demyelinating features are suggestive of hereditary demyelination, whereas difference between nerves and segments of the same nerve favor acquired demyelination. Finally, neuropathy is classified into mononeuropathy commonly due to entrapment or trauma; mononeuropathy multiplex commonly due to leprosy and vasculitis; and polyneuropathy due to systemic, metabolic or toxic etiology. Laboratory investigations are carried out as indicated and specialized tests such as biochemical, immunological, genetic studies, cerebrospinal fluid (CSF) examination and nerve biopsy are carried out in selected patients. Approximately 20% patients with neuropathy remain undiagnosed but the prognosis is not bad in them.

Peripheral neuropathy is a general term that indicates any disorder of the peripheral nervous system. It is a common neurological disorder, with variable presentation and numerous causes. The broad definition of peripheral neuropathy includes all types of diseases associated with the peripheral neurons system; hence, there is a need to subclassify this disorder, and the clinical approach has to be sequential and logical for a cost-effective management. In this review, we highlight well-founded principles of clinical diagnosis and investigations that are useful for both general physicians and specialists.

## Epidemiology

The overall prevalence of peripheral neuropathy is 2.4%; however, it increases to 8% in individuals aged above 55 years.[1] These figures do not include traumatic peripheral neuropathies; therefore, the total burden of peripheral neuropathies is likely to be higher. In the developed world, diabetes mellitus is the most common cause of this disease. In a Dutch population-based study to estimate the incidence of neuropathic pain among 362,693 individuals, mononeuropathy (4.3/1000/year) and carpal tunnel syndrome (2.3/1000/year) were the most frequent incidences followed by diabetic peripheral neuropathy (0.72/1000/year) and post-herpetic neuralgia (0.42/1000/year).[2] In India and other developing countries, the incidence of diabetes has increased; therefore, the incidence of diabetic neuropathy is also likely to increase. In global terms, leprosy continues to be a major cause of neuropathy and is a particular problem in developing countries. *Campylobacter jejuni* and a number of viral infections are widely prevalent and result in peripheral nerve demyelination and/or axonal neuropathies, which are important problems in China, India and other regions. Other important causes of peripheral neuropathy are nutritional deficiency, alcoholism, vasculitis, systemic disease and exposure to toxins. There are over 100 causes of neuropathy.[3] The clinician has to determine the underlying treatable cause, which can be achieved by adopting a systematic approach. Diagnostic algorithm for peripheral neuropathy has been published previously.[4] It was believed that in 50% of the cases, the etiology of neuropathy remained undiagnosed;[5] however, several large series have shown that after intensive investigations, only approximately 20% of the cases remain undiagnosed and these tend to have a good prognosis.[6]

## Clinical Approach

The initial step is to confirm whether the signs and symptoms are related to peripheral nerve dysfunction. Occasionally, the patient with neuropathy may present with multiple pathologies. Spinal cord disease is the most common differential diagnosis in patients with neuropathic symptoms. In some patients with myelopathy, the sensory symptoms are present with few clinical signs; the classic signs of lower motor neuron involvement may be absent, simulating peripheral neuropathy. Patients with lacunar stroke may rarely present with sensory loss in median or ulnar nerve distribution.[7] Although patients with spinal canal stenosis present classically with neurogenic claudication, in advanced stage, they may be associated with persistent symptoms and the condition may be confused with peripheral neuropathy.[8]

In elderly patients, often there is a coexistence of cervical spondylotic myelopathy with late onset predominantly sensory axonal neuropathy. Similarly, spondylotic radiculopathy may occur with upper limb entrapment neuropathies, and the coexisting pathologies should be carefully diagnosed. Neuropathy may also occur with CNS involvement in vitamin B_12_ deficiency, adrenomyeloneuropathy and acanthocytosis.

The peripheral nerves comprise sensory, motor and autonomic fibers, which have different lengths, diameters, conduction characteristics and specialized functions. Their involvement therefore results in diverse symptoms, signs and EDx features. Focusing on these symptoms is helpful in the diagnosis of peripheral neuropathy.

## History

Occasionally, simple history such as funny feet, unevenly worn shoes, and childhood clumsiness are important clues to a long-standing illness well beyond the presenting symptoms. The duration of symptoms is important in categorizing neuropathy into acute (<4 weeks), subacute (4–12 weeks) and chronic (>12 weeks). Vasculitis results in hyperacute mononeuropathies usually occurring by 24–72 h. Acute inflammatory demyelinating polyradiculoneuropathy (AIDP) by definition peaks by 4 weeks of onset, and a progression beyond 8 weeks suggests chronic inflammatory demyelinating polyradiculoneuropathy (CIDP). The time course study helps in limiting the diagnosis for acute and demyelinating conditions, which have different diagnostic and therapeutic approaches. The diagnostic criteria of AIDP are presented in Table 1[9] and those of CIDP in Table 2.[10] Sensory symptoms are usually the presenting symptoms of neuropathy and include positive (burning, pain, walking on cotton wool, band-like sensation on feet or trunk, stumbling, tingling, pins and needles) and negative symptoms (numbness, loss of sensation) in hands and feet. Motor symptoms include weakness and patient may complain of difficulty in turning keys in locks, unfasten button and opening bottles and jars. In the early stage, weakness in peripheral neuropathy is distal; however early proximal weakness is a feature of inflammatory neuropathy and porphyric neuropathy.

**Table 1:** Diagnostic criteria for Guillain Barre Syndrome (ref)

**Features required for diagnosis**
Progressive weakness of both legs and arms
Areflexia
**Clinical features supportive of diagnosis**
Progression over days to 4 weeks
Relative symmetry or signs
Mild sensory symptoms and signs
Cranial nerve involvement (bifacial palsies)
Recovery beginning 2–4 weeks after progression ceases
Autonomic dysfunction
Absence of fever at onset
**Laboratory features supportive of diagnosis**
Elevated CSF protein with <10 cells/µl
EDx features of nerve conduction slowing or block.[*]

* Features supporting an axonal process are seen in acute motor axonal neuropathy and acute motor sensory axonal neuropathy

Adapted from Ashbury, A. K., and Cornblath, D. R., 1990, Assessment of current diagnostic criteria for Guillain-Barré syndrome, Ann Neurol, vol. 27, suppl., pp. S21-S24.

**Table 2:** Diagnostic criteria of chronic inflammatory demyelinating poly neuropathy (Ref)

The diagnosis of CIDP is based on a combination of clinical features, nerve conduction studies, spinal fluid analysis and in selected cases, nerve biopsy.
Clinical criteria:
1- *Inclusion criteria*
A) Typical CIDP
Chronically progressive, stepwise or recurrent symmetric proximal and distal weakness and sensory dysfunction of all extremities, developing over at least two months; cranial nerves may be affected Absent or reduced tendon reflexes in all extremities
B) Atypical CIDP
One of the following but otherwise as in A (tendon reflexes may be normal in unaffected limbs)
Predominantly distal weakness (distal acquired demyelinating symmetric; DADS) Pure motor or sensory presentations (and possibly autonomic) Asymmetric presentations (multifocal acquired demyelinating sensory and motor (MADSAM), Lewis-Sumner syndrome) Asymmetric presentations (multifocal acquired demyelinating sensory and motor (MADSAM), Lewis-Sumner syndrome) Focal presentations (e.g., involvement of the brachial plexus or of one or more peripheral nerves in one upper limb) Central nervous system involvement (may occur in otherwise typical CIDP)
2- *Exclusion criteria*
Diphtheria, drug or toxin exposure, hereditary demyelinating neuropathy, presence of sphincter disturbance, multifocal motor neuropathy, antibodies to myelin associated glycoprotein
EDx criteria
I) *Definite*
At least 50% prolongation of motor distal latency above the upper limit of normal values in two nerves, **or** At least 30% reduction of motor conduction velocity below the lower limit of normal values in two nerves, **or** At least 20% prolongation of F-wave latency above the upper limit of normal values in two nerves (>50% if amplitude of distal negative peak CMAP <80% of the lower limit of normal values) **or** Absence of F-waves in two nerves if the amplitude of distal negative peak CMAP at least 20% of lower limit of normal values + at least one other demyelinating parameter in at least one other nerve **or** Partial motor conduction block: at least 50% reduction in the amplitude of the proximal negative peak CMAP if distal negative peak CMAP is at least 20% of lower limit of normal values in two nerves or in one nerve + at least one other demyelinating parameter in at least one other nerve **or** Abnormal temporal dispersion (>30% duration increase between the proximal and distal negative peak CMAPs) in at least two nerves **or** Distal CMAP duration (interval between onset of the first negative peak and return to baseline of the last negative peak) of at least 9 ms in at least 1 nerve + at least 1 other demyelinating parameter in at least 1 other nerve.
2) *Probable:*
At least 30% reduction in the amplitude of the proximal negative peak CMAP, excluding the posterior tibial nerve, if distal negative peak CMAP at least 20% of the lower limit of normal values in two nerves or in one nerve + at least one other demyelinating parameter in at least one other nerve
3) *Possible:* As in I but in only one nerve
Supportive criteria
Elevated CSF protein with cell counts <10/mm3 Magnetic resonance imaging showing gadolinium enhancement and/or hypertrophy of the cauda equina, lumbosacral or cervical nerve roots, or the brachial or lumbosacral plexuses Nerve biopsy showing unequivocal evidence of demyelination and/or remyelination in >5 fibers by electron microscopy or in >6 of 50 teased fibers Clinical improvement following immunomodulatory treatment
Diagnostic categories
*Definite CIDP:* Clinical criteria 1 A **or** B and 2 and EDx criteria 1; **or** Probable (electrophysiology) CIDP + at least 1 Supportive criterion or Possible (electrophysiology) CIDP + at least 2 Supportive criteria *Probable CIDP:* Clinical criteria 1 A **or** B and 2 with EDx criteria 2;or Possible (electrophysiology) CIDP + at least 1 Supportive criterion *Possible CIDP:* Clinical criteria 1 A **or** B and 2 with EDx criteria 3

CIDP - Chronic inflammatory demyelinating polyneuropathy

Autonomic symptoms such as postural hypotension, impotence, sphincter disturbance, diarrhea, constipation, dryness or excessive sweating point to the involvement of small myelinated or unmyelinated nerve fibers.

## Symptoms and Topography

Precise details regarding the site and character of sensory symptoms are helpful in localizing and characterizing the neuropathy as in meralgia paresthetica and carpal tunnel syndrome. Distal dying back axonopathies have a characteristic length-dependent pattern of the evolution of symptoms, which are usually symmetrical and affect feet, hand and trunk. Demyelinating neuropathies may also have a length-dependent pattern of sensory evolution because in a diffuse process, longer fibers have a greater likelihood of being blocked. In a multisegmental pattern of sensory involvement, including trunk, suggests dorsal root gangioneuropathies, as observed in Sjogren's syndrome-associated neuropathy.

Pain, loss of temperature sensation and autonomic symptoms are the features of small fiber neuropathy. Ataxia in the dark or on eye closure is suggestive of large fiber involvement. These sensory patterns do not localize the lesion even to the peripheral nerve but provide a notion regarding the involvement of fiber type and narrow down the diagnostic possibilities.

## Examination

**Figure 1:**
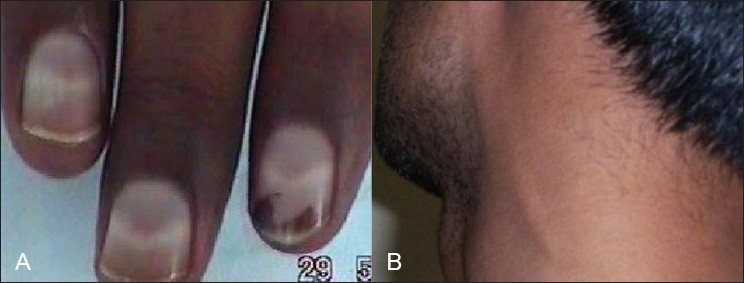
(A) Photograph of a patient with arsenic neuropathy shows Mee's line (B) photograph showing great auricular nerve thickening

**Figure 2:**
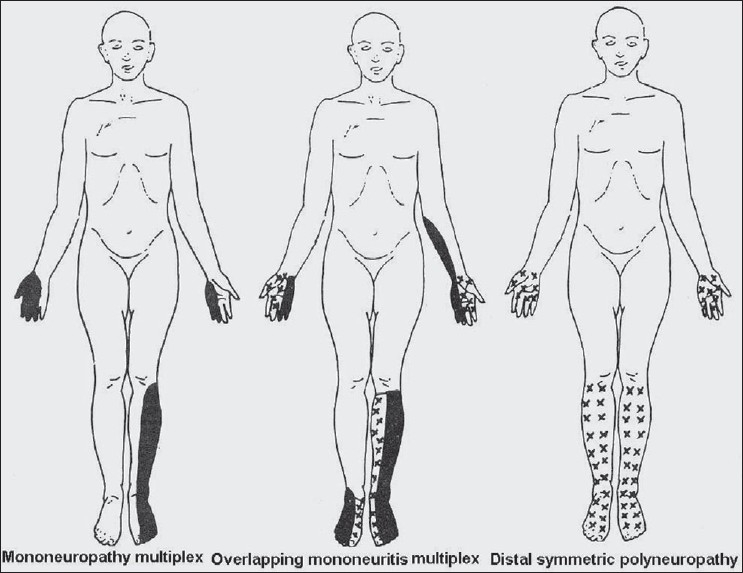
Schematic diagram shows topography of deficit inmononeuropathy multiplex, overlap neuropathy and distal symmetrical polyneuropathy

A number of important diagnostic clues can be identified on general examination, but often require a return visit to the patient. Umbilical keratomas of Fabry's disease, Mee's lines in arsenic [Figure 1] and thallium poisoning, orange tonsils in Tangier's disease are rare diagnostic opportunities available to well-trained clinicians. Maculoanesthetic patches with thickened nerves are the diagnostic characteristics of leprosy [Figure 2]. On cranial nerve examination, anosmia is a feature of Refsum's disease and vitamin B_12_ deficiency; impaired pupillary light reflex may indicate parasympathetic involvement and prompt a detailed search for dysautonomia which may occur in diabetic neuropathy and GB syndrome.

External ophthalmoplegia is a feature of Miller Fisher syndrome, facial weakness of GB syndrome and trigeminal sensory loss of Sjogren's syndrome and lower cranial nerve palsy with gynecomastia of Kennedy's syndrome. The presence of musculoskeletal abnormality such as pes cavus, high-arched feet and mutilation suggest hereditary neuropathy.

Muscle power testing in the context of nerve and root distribution is crucial. Difficulties arise when multiple mononeuropathies become confluent, thus making the differentiation from polyneuropathy difficult. In such a situation, electrodiagnostic (EDx) studies are invaluable. Tendon reflexes are important in the diagnosis of neuropathies. Distal reflex loss manifesting with absent ankle reflex but preserved reflexes elsewhere are characteristic of length-dependent axonopathies. In acquired demyelinating neuropathies, reflex loss is usually generalized as in CMT I.

The sensory examination is best performed by testing modalities that subserve large fibers (vibration and joint position) and small fiber (pinprick, pain and temperature) in conjunction with consideration for both focal and length-dependent features since it can provide important diagnostic clues to the likely cause.

## Characteristics of the topography of neuropathy

**Figure 3:**
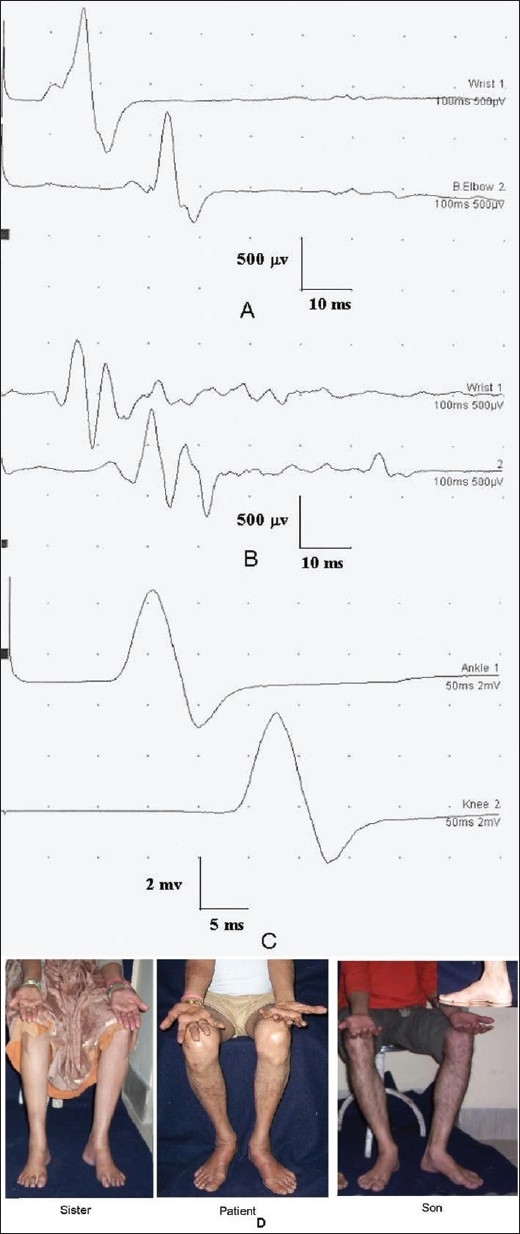
Nerve conduction study of a 52-year-old male with hereditary motor sensory neuropathy showing slowing of conduction velocity and reduced CMAP in (A) ulnar (16.8 m/s; 0.9 mV and 0.8) and median (22 m/s; 0.5 and 0.6 mv) motor conductions. His peroneal and sural conductions were unrecordable. (C) Peroneal conduction study of his son who was asymptomatic showed slowing of conduction velocity (23.6 m/s). (D) Photograph of the patient and his sister and son suggesting AD in heritance. There was wasting and weakness of small muscles of hands and feet of the patient and high-arched feet of the sister and son (inset)

The topography of involvement is helpful in the diagnosis of peripheral neuropathy. On the basis of topography, the neuropathies can be categorized into mononeuropathy, mononeuropathy multiplex and polyneuropathy [Figure 3].

Mononeuropathy refers to single peripheral nerve involvement and usually occur due to trauma, compression or entrapment. The common entrapment neuropathies are carpal tunnel syndrome, ulnar nerve entrapment at the elbow and peroneal nerve entrapment at the head of the fibula. EDx are invaluable in the diagnosis of mononeuropathies to localize and assess the severity of neuropathy. Mononeuropathy especially at an entrapment site are often considered an isolated phenomenon possibly related to pregnancy, thyroid disease or occupation. However, neuropathy may occur as a feature of a more generalized disorder such as hereditary neuropathy with liability to pressure palsy or amyloidosis. Mononeuropathy occurring outside entrapment sites should be carefully investigated. The possible causes of focal or multifocal neuropathies are considerably smaller than generalized neuropathies.

Mononeuropathy multiplex refers to the involvement of multiple, separate noncontiguous peripheral nerves either simultaneously or sequentially. Sometimes, mononeuropathy may aggregate resembling polyneuropathy. Focusing on the pattern of early symptoms facilitates accurate diagnosis. Mononeuropathy multiplex occurs most commonly due to leprosy and systemic vasculitis (polyarteritis nodosa, Churg-Strauss syndrome, rheumatoid arthritis, Sjogren's syndrome) and requires urgent diagnosis [Table 3]. In suspected vasculitis, sural or superficial peroneal nerve biopsy is often helpful.

**Table 3:** Causes of focal-multifocal neuropathies

1.	Entrapment neuropathy: Carpal tunnel syndrome, ulnar nerve at the elbow, common peroneal at the fibular head
2.	Endocrinal: diabetes mellitus, myxedema, acromegaly
3.	Amyloidosis
4.	Hereditary neuropathy susceptible to pressure palsy
5.	Vasculitis
6.	Multifocal motor neuropathy with conduction block

Distal symmetrical polyneuropathy is the most common variety of neuropathy. The nerve fibers are affected in a length-dependent pattern; toes and soles are affected first and hands later. A majority of these cases occur due to metabolic, toxic or systemic disorders.

## Extent of sensory versus motor involvement

Some neuropathies are purely motor (multifocal motor neuropathy with conduction block) or sensory (subacute sensory neuropathy caused by paraneoplastic or autoimmune dorsal root ganglionopathies) although a majority of cases are mixed if not symptomatically then on clinical examination or EDx studies. Multifocal acquired demyelinating sensory and motor neuropathy (MADSAM; Lewis Summer syndrome) needs consideration in appropriate clinical setting. In contrast, sensory involvement should generally exclude motor neuron disease; similarly, motor involvement should exclude dorsal root gangliononeuropathy.

Small fiber neuropathy manifests with dysautonomia such as sweating, pupillary, cardiovascular, gastrointestinal and micturition disturbances. These features may also be found in GB syndrome, which is a large fiber neuropathy. The causes of small fiber neuropathy are summarized in Table 4.

**Table 4:** Causes of small fiber neuropathy

•	Diabetes
•	Amyloidosis
•	Fabry's disease
•	Tangier's disease
•	Hereditary sensory and autonomic neuropathy
•	Sjogren's syndrome
•	Chronic idiopathic small fiber sensory neuropathy

Another important question is to decide whether the neuropathy is predominantly axonal or demyelinating. This question requires the use of EDx since it cannot be determined with certainty only on the basis of clinical evaluation. The helpful clinical clues include the following: (1) Widespread reflex loss, including muscles that are not particularly weak or wasted, favor demyelinating neuropathy. (2) Selective loss of ankle reflex in the presence of distal wasting and weakness is characteristic of axonopathy if accompanied by distal sensory loss. The causes of axonal neuropathy are summarized in Table 5.

**Table 5:** Causes of chronic axonal neuropathies

1.	Drug and toxin: Alcohol, vincristine, phenytoin, organophosphate, statins, metronidazole, dapsone
2.	Infection: Leprosy, HIV, Borreliosis
3.	Connective tissue: Sjogren's syndrome, systemic lupus erythematosus, rheumatoid arthritis
4.	Metabolic: Diabetes, chronic renal failure
5.	Paraneoplastic: Carcinoma of the lung and ovary.
6.	Inherited: CMT 2 and CMT X
7.	Vitamin deficiency: B_12_, Folic acid, Vitamin E
8.	Endocrine: Hypothyroidism
9.	Paraproteinemia: Myxedema, Waldenstorm's disease, Benign monoclonal gammopathy

The largest and important group is the mixed motor sensory neuropathy group. In these patients, careful investigation of the history of systemic illness and family history is important.

## EDx tests

For the evaluation of peripheral neuropathy, nerve conduction study of sensory and motor nerves, late responses (F response and H reflex) and needle electromyography (EMG) are performed. Conduction block refers to a decline in the compound muscle action potential exceeding 20% on proximal stimulation compared to that on distal stimulation. The slowing of nerve conduction velocity, prolongation of terminal latency, temporal dispersion and conduction block are consistent with demyelinating neuropathy. Uniform demyelination favors inherited neuropathy. On the other hand, findings with difference between nerves and segments of the same nerve are more in favor of acquired demyelination.[11,12]

In axonal neuropathy, there is mild slowing of nerve conduction due to a fall out of large-diameter axons, whereas the remaining axons may have normal nerve conduction. The other evidence of axonal neuropathy is reduced CMAP amplitude and fibrillations on EMG. Sensory nerve action potentials and sensory conduction velocities are reduced in both axonal and demyelinating neuropathies.

For the interpretation of nerve conduction studies, the age of the patient needs to be considered. Normal nerve conduction velocity is half the adult value in infant, reaches the adult range by 3–5 years of age, and may decline in the elderly. For the interpretation of results, the temperature of the limb should be taken into account. The conduction velocity changes by 2.4 m/s for each degree change in centigrade from 29 to 38 °C.[13,14]

Nerve conduction and EMG studies are uncomfortable for the patient despite the neurophysiologist's best efforts. It takes 30–60 min of neurophysiologist's precious time. The EDx tests do not replace or substitute clinical evaluation but supplement it and it is more difficult to obtain cooperation from patients for EDx than for clinical examination. It is easier to localize significant weakness or sensory loss than the mild ones, and the same holds true for the EDx study. EMG and nerve conduction studies are operator dependent and proper standardization of the technique and generating laboratory' control values are essential pre-requisites for proper interpretation of the results.

EMG and nerve conduction studies are useful in localizing peripheral nervous system deficit found on clinical examination. One should be able to frame an answerable question before commencing EMG and nerve conduction studies. EDx tests do not provide information regarding the cause of neuropathy but localize the lesion more precisely than on clinical examination alone. EDx provides invaluable help in the diagnosis of acute and chronic inflammatory demyelinating polyradiculoneuropathy, multifocal motor neuropathy with conduction block and entrapment neuropathy (carpal tunnel syndrome, ulnar neuropathy at elbow and peroneal neuropathy at the fibula).

Nerve conduction studies categorize neuropathy according to distribution (mono neuropathy, mononeuropathy multiplex or generalized), axonal vs demyelinating and small vs large fiber neuropathy. A majority of patients investigated have distal symmetrical axonopathies, which occur mostly due to diabetes or alcohol and have a simple diagnosis. In such a situation, in 50% cases, EDx does not contribute substantially to the diagnosis especially if the duration of neuropathy is more than 6 weeks.[15] This study suggested that demyelinating neuropathies can be diagnosed on clinical grounds. Although the role of EDx in chronic neuropathies is disputed, this is not the case in acute neuropathies, asymmetrical neuropathies, mononeuropathies or in any severe disabling neuropathy. The differential diagnosis will include acute inflammatory demyelinating polyneuropathy, chronic inflammatory demyelinating polyneuropathy and vasculitic neuropathy. EDx in such cases helps in diagnosis, treatment decision or strengthens the need for nerve biopsy. Any patient with peripheral neuropathy that is not entirely typical for the putative cause should be considered for EDx.[16] Small fiber neuropathy requires other investigations, since it cannot be evaluated by EDx. In the early stage, the GB syndrome may not show any changes in EMG and nerve conduction study.

Some researchers recommend investigation of common causes of peripheral neuropathy before undertaking EDx tests,[15] but this conclusion is not supported by the evidence.[17–20] EDx studies are sensitive, specific, and validated measures of peripheral neuropathy. The EDx tests enable the determination of the type (demyelinating vs axonal), and confirm the topography of neuropathy, i.e., mononeuropathy, mononeuropathy multiplex or polyneuropathy. The EDx finding in common focal neuropathies are summarized in Tables 6 and 7.

**Table 6:** Electrodiagnostic findings in localizing upper limb focal neuropathies

Nerve	Site of lesion	Focal slowing	Change in SNAP/CMAP	NCV change	Comments
Common					
Median	CTS	+++	+++	+	EMG not needed
Ulnar	Elbow	+	++++	++
Uncommon					
Radial	Upper arm	+	++	+++
Axillary	Humeral head	NA	NA	+++
Ulnar	Wrist	+	++	+++	Other ulnar studies
Long thoracic	Not clear	NA	NA	++	Pneumo-thorax

CTS = carpal tunnel syndrome, NA = not available, SNAP = sensory nerve action potential, CMAP = compound muscle action potential, NCV = nerve conduction velocity

**Table 7:** Electrodiagnostic studies used in localizing lower limb neuropathies

Nerve	Site of lesion	Focal slowing	Change in SNAP/CMAP	NCV change	Comments
Common					
LFCN of thigh	Inguinal le	NA	+/−	NA	EDx linked
Common Peroneal	Fibular head	++	+++	++
Uncommon					
Sciatic	Pelvic, thigh	NA	++	+++	Rule out LS plexopathy
Tibial ankle	Tarsal tunnel	+	++	++

LFCN = left femoral cutaneous nerve, NA = not available, SNAP = sensory nerve action potential, CMAP = compound muscle action potential, NCV = nerve conduction velocity

## Laboratory tests

The clinical and EDx evaluations should be followed by the first line laboratory tests as listed below.

First-line screening test for neuropathy

- Blood count, ESR
- Blood sugar
- Liver and renal function tests
- Serum vitamin B_12_
- Paraprotein levels
- Thyroid function tests
- Vasculitis profile

If history, examination, EDx and the abovementioned investigation do not reveal a diagnosis, one should revise family history and examine the family members. Further tests are performed on the basis of clinical clues. Vasculitis restricted to peripheral nerves may require nerve biopsy, but the yield of nerve biopsy in distal chronic idiopathic symmetrical polyneuropathy is very low. However, if the neuropathy is of recent onset or progressive, nerve biopsy should be performed. Anti-Hu antibody is clearly associated with paraneoplastic neuropathy[21] and undisclosed malignancy usually of the lung and ovary.

Cerebrospinal fluid (CSF) is useful in CIDP, AIDP and chronic immune-mediated axonal neuropathies where the levels of CSF protein are elevated. Significant pleocytosis should raise the suspicion regarding other acute inflammatory neuropathies such as Borrelia, sarcoidosis or human immunodeficiency virus (HIV).

Genetic testing is available for a number of hereditary neuropathies such as CMT I–IV and CMT X. Since the role of genetic testing is evolving, conducting a battery of tests for a condition for which no specific treatment is presently available should be restricted. In a patient with two generations of neuropathy with male to male transmission, and uniform conduction showing PMP22 duplication test should be obtained. If normal, PMP22 and MPZ DNA sequencing are recommended. If there is no male to male transmission and CMT 1A duplication, screening for connexin 32 should be undertaken. Because of high spontaneous mutation rate, the diagnosis of CMT 1 A and HNPP should be considered even in the case of a negative family history. CMT 1A patients are susceptible to severe reaction to vincristine and other chemotherapeutic drugs; therefore, it is important to rule out CMT 1A in neuropathy patients in whom cancer chemotherapy is planned. For axonal neuropathy, DNA sequencing tests are available for Cx32, MPZ, NF-L and MNF2. In children with severe hereditary demyelinating neuropathy, PMP22 duplication test followed by DNA sequencing of PMP22, MPZ, EGR2 and periaxin should be considered.[22] Antiganglioside antibodies are elevated in patients with multifocal motor neuropathy with conduction block (MMN-CB) in approximately 50% of the patients. Its absence however does not exclude the diagnosis of MMN-CB but the presence is helpful. Anti-GQ1b IgG antibodies are a marker of Miller Fisher syndrome. In motor axonal variant of GB syndrome, anti-GM1 and anti-GDI antibodies are found in 50% cases.

## Nerve biopsy

Sensory nerve biopsy is an established diagnostic procedure, but should be performed in the center where facilities for electron microscopy, teased fiber technique and immunohistochemistry are available.[23] Biopsy only to confirm the presence of neuropathy is not necessary. With the advent of genetic testing, the need for biopsy remains the primary method of establishing vasculitic neuropathy when histology is not available from elsewhere. Combined nerve and muscle biopsy has been recommended to improve the diagnostic yield.[24] In a prospective study on 50 nerve biopsies in consecutive patients, the management was altered in 60% and diagnosis altered in 14%. Biopsy was considered to cause persistent pain in 33% patients.[23]

Nerve biopsy can be helpful in the diagnosis of CIDP in which the presence of inflammatory cells or macrophage-mediated demyelination on electron microscopy is diagnostic. Unfortunately many patients with CIDP do not have inflammatory cells in their sensory nerves, and biopsy is probably unnecessary if EDx is suggestive and clinical features are typical.[25] In majority of cases, the result of quantitative analysis by using light microscopy was similar in CIDP and chronic idiopathic axonal neuropathy; hence, sural nerve biopsy is of limited value in these conditions.[26] The yield of biopsy in the diagnosis of chronic axonal neuropathies is very small and not justified when vasculitis is unlikely. Patients with axonal neuropathy simulating the axonal form of Charcot Marie Tooth can occasionally turn out to have amyloid, especially if there is the involvement of small fibers. Diagnosis can be made by transthyretin mutations without resorting to biopsy, but biopsy is helpful in the absence of a genetic marker.[27]

Small fiber neuropathies cannot be diagnosed by EDx studies and require quantitative sensory testing to define cold, hot and pain threshold. Routine sensory conduction studies evaluate only fast conducting fibers, which may be normal in selective small fiber and autonomic neuropathies. In such patients, quantitative sensory testing evaluating pain, cold and hot threshold, tests of sudomotor functions and skin biopsy with intraepidermal nerve fiber density and quantification of protein gene product 9.5, which is a pan axonal marker may be performed. These tests do not provide specific etiological diagnosis and are seldom abnormal in isolation. The results of skin biopsy may be abnormal in 10% patients with normal sudomotor functions.[28]

Autonomic neuropathies should be suspected on the basis of the features of orthostatic hypotension, hyperhydrosis, genitourinary symptoms (impotence, nocturia, retention of urine), gastrointestinal (constipation, postprandial fullness, diarrhea) and worsening of the symptoms on bed rest, alcohol, hot bath, exercise and hyperventilation. Bedside autonomic tests include blood pressure response to standing or vertical tilt (Normal fall, <20/10 mmHg), heart rate response to standing (increase, 11–90 beats/min; 30:15 ratio ≥ 1.04), isometric exercise (normal increase in diastolic blood pressure, 15 mmHg), heart rate variation with respiration (normal, ≥15 beats/min, inspiratory expiratory ratio 1.2), valsalva ratio (N≥1.4), cold pressure test (after 1 min systolic blood pressure, 15–20 mmHg/diastolic 10–15 mmHg), sweat test, axon reflex (piloerection, sweating), pupillary tests, Schirmer's test (15 mm after 5 min). Autonomic function can also be evaluated by sympathetic skin response.

## Balanced approach

Neurologists fall into two polar groups: (A) Pragmatists aiming at minimal possible investigations to solve the clinical problem. They usually work in secondary centers. (B) Completists who aim to eliminate every possibility, however remote it may be, even if it may not have a therapeutic application. Usually, they work as experts in tertiary centers. The pragmatists are at a risk of missing the rare conditions, particularly if these mimic common conditions. On the other hand completists are at a risk of misdiagnosis as they may be misled by false-positive results.[16] It is important to have a balanced view and follow the clinical and investigative clues and possibility of therapeutic potential of the likely diagnosis.[29] It is recommended to do the right thing and not everything.
